# MicroRNA-150 Predicts a Favorable Prognosis in Patients with Epithelial Ovarian Cancer, and Inhibits Cell Invasion and Metastasis by Suppressing Transcriptional Repressor ZEB1

**DOI:** 10.1371/journal.pone.0103965

**Published:** 2014-08-04

**Authors:** Minfei Jin, Zujing Yang, Weiping Ye, Hongling Xu, Xiaolin Hua

**Affiliations:** Department of Obstetrics and Gynecology, Xinhua Hospital, Shanghai Jiaotong University School of Medicine, Shanghai, China; King Faisal Specialist Hospital & Research center, Saudi Arabia

## Abstract

MicroRNA (miR)-150 has been reported to be dramatically downregulated in human epithelial ovarian cancer (EOC) tissues and patients’ serum compared to normal controls. This study aimed to investigate clinical significance and molecular mechanisms of miR-150 in EOC. In the current study, quantitative real-time PCR analysis showed that miR-150 was significantly downregulated in human EOC tissues compared to normal tissue samples. Then, we demonstrated the significant associations of miR-150 downregulation with aggressive clinicopathological features of EOC patients, including high clinical stage and pathological grade, and shorter overall and progression-free survivals. More importantly, the multivariate analysis identified miR-150 expression as an independent prognostic biomarker in EOC. After that, luciferase reporter assays demonstrated that Zinc Finger E-Box Binding Homeobox 1 (ZEB1), a crucial regulator of epithelial-to-mesenchymal transition (EMT), was a direct target of miR-150 in EOC cells. Moreover, we found that the ectopic expression of miR-150 could efficiently inhibit cell proliferation, invasion and metastasis by suppressing the expression of ZEB1. Furthermore, we also observed a significantly negative correlation between miR-150 and ZEB1 mRNA expression in EOC tissues (rs = –0.45, P<0.001). In conclusion, these findings offer the convincing evidence that aberrant expression of miR-150 may play a role in tumor progression and prognosis in patients with EOC. Moreover, our data reveal that miR-150 may function as a tumor suppressor and modulate EOC cell proliferation, and invasion by directly and negatively regulating ZEB1, implying the re-expression of miR-150 might be a potential therapeutic strategy for EOC.

## Introduction

Epithelial ovarian cancer (EOC) represents the fifth most lethal gynecologic malignancy and originates from the ovarian surface, inclusion cysts in the ovarian parenchyma, or from the nearby distal fallopian tube epithelium [1]. Because there are few effective biomarkers and therapies, EOC is an aggressive disease which causes estimated 125,000 deaths all over the world annually [2]. Five-year survival of patients with EOC is critically dependent on the clinical stage at patients’ diagnosis; if diagnosed and treated while localized (stages I and II), the 5-year survival rates can reach over 90% [3]. However, most EOC patients are diagnosed as advanced disease (stages III and IV) where the 5-year survival is only 30∼40% [4]. These data suggest that the clinical outcome of EOC patients may be significantly higher with early diagnosis, however, currently there is no non-invasive method to accurately detect EOC at an early stage. Given this scenario, the development of novel and efficient diagnostic and prognostic molecular biomarkers for EOC is needed.

MicroRNAs (miRNAs), as a novel class of small noncoding single-stranded RNAs, have recently been demonstrated to regulate gene expression post-transcriptionally through base pairs complementary to the binding sites on the 3′-UTR of the target mRNA, leading to target mRNA cleavage or translational repression [5]. By binding with their target genes, miRNAs are implicated into various biological processes, including cell proliferation, apoptosis as well as cell differentiation [6]. A growing evidence has reported that miRNAs may play essential roles in cancer cell invasion and metastasis. Especially in EOC, Yeh et al. [7] indicated the downregulation of miRNA-138 in the highly invasive cells, and its functioning as an inhibitor of cell migration and invasion; Wang et al. [8] found that miR-182 may act as an oncogenic miRNA and promote cancer cell growth, invasion, and chemoresistance by targeting PDCD4 in EOC cells; Wu et al. [9] suggested that miR-145 may modulate EOC growth and invasion by suppressing p70S6K1 and MUC1, functioning as a tumor suppressor. These previous studies provided initial clues for the contributions of loss or gain function of specific miRNAs to tumorigenesis and cancer progression of EOC.

MiR-150, localized on chromosome 19q13, has been indicated as a hematopoietic-specific miRNA in malignant lymphoma and has been observed to be significantly downregulated in tumor cells relative to healthy cells [10]. The abnormal expression of miR-150 has also been found in other various solid tumor tissues, such as lung cancer, gastric cancer, colorectal cancer, endometrial cancer, EOC, and pancreatic cancer [11]–[16]. Especially, Vang et al. [14] found that miR-150 displayed low expression in most primary EOC tissues; Shapira et al. [15] also reported that miR-150 showed at least a 10-fold decrease in expression in pre-surgical plasma samples from women diagnosed with EOC compared with plasma samples from women without a known pelvic mass (healthy controls). These findings imply a possible role of miR-150 in human EOC, which prompted us to identify and functionally validate miR-150-associated clinical significance and molecular mechanisms in EOC.

## Materials and Methods

### Patients and Tissue Samples

Clinical samples were obtained from Xinhua hospital, Shanghai Jiaotong University School of Medicine, China. Written informed consent was obtained from all patients and the study was approved by the Ethics Committee of Xinhua hospital, Shanghai Jiaotong University School of Medicine, China.

For quantitative real-time PCR assay, 100 EOC tissue specimens and 10 normal ovarian tissue specimens were snap-frozen in liquid nitrogen and stored at –80°C following surgery which were performed in the Department of Obstetrics and Gynecology, Xinhua hospital, Shanghai Jiaotong University School of Medicine from December 2006 to January 2008. All 10 normal ovarian tissues were obtained from women who underwent hysterectomies for benign disease. All EOC patients were treated without preoperative radiotherapy, chemotherapy, or hormonal therapy. Surgical staging was established according to the International Federation of Gynecology and Obstetrics (FIGO) system. The clinical features of 100 EOC patients were summarized in Table 1.

**Table 1:** Association of microRNA-150 (miR-150) expression with clinicopathological features of epithelial ovarian cancer tissues.

Features	No. of patients	miR-150 expression (n, %)		P
		Low	High	
**Age**				
<56	40	20 (50.00)	20 (50.00)	NS
≥56	60	38 (63.33)	22 (36.67)	
**Clinial stage**				
I∼II	20	6 (30.00)	14 (70.00)	0.005
III∼IV	80	52 (65.00)	30 (35.00)	
**Pathological grade**				
1∼2	30	13 (43.33)	17 (56.67)	0.01
3	70	45 (64.29)	25 (35.71)	
**Histological type**				
Serous	85	50 (58.82)	35 (41.18)	NS
Non-serous	15	8 (53.33)	7 (46.67)	
**Residual tumor after surgery**				
<1 cm	60	33 (55.00)	27 (45.00)	NS
≥1 cm	40	25 (62.50)	15 (37.50)	

Note: ‘NS’ refers to the difference without statistical significance.

Regular follow-ups (range: 2.08–119.06 months, 62.86 months in median, 62.66 months in average) were performed after the treatment of all 100 EOC patients enrolled in the current study, with their survival time, date of death and date of last follow-up being recorded. Overall survival (OS) was defined as the time interval from the date of diagnosis at our center to the date of death or the last follow-up. Progression-free survival (PFS) was defined as the time interval from diagnosis at our center to progressive disease, death of any other cause than progression, or a second primary cancer.

### Cell culture

Human ovarian serous cystic adenocarcinoma cell line OVCAR3 and human serous papillary cystic adenocarcinoma cell line SKOV3 were purchased from American Tissue Type Collection (Manassas, VA). Both cell lines are suitable transfection hosts. All cells were cultured in RPMI-1640 medium supplemented with 10% fetal calf serum (GIBCO) in a humidified atmosphere of 5% CO_2_ at 37°C.

### RNA and miRNA extraction

For mRNA quantification, total RNAs from cell lines and tissues were extracted using Trizol reagent (Invitrogen) according to the manufacturer’s instructions. For miRNA quantification, total miRNA was extracted from cell lines and tissues using the mirVana miRNA Isolation Kit (Ambion, Austin, TX, USA) according to the manufacturer’s instructions.

### Quantitative real-time PCR assay

For mRNA and miRNA quantifications, quantitative real-time PCR assay was performed to respectively detect the expression levels of miR-150 and ZEB1 in cell lines and tissues. Briefly, 10 µg of small RNA and 20 µg of total RNA was subjected to reverse transcription. The cDNA was used for the amplification of mature miR-150, ZEB1 and the endogenous controls, U6 and β-actin, by PCR. The PCR primers used were as follows: miR-150 forward, 5′-CAG TAT TCT CTC CCA ACC CTT GTA-3′ and reverse 5′-AAT GGA TGA TCT CGT CAG TCT GTT-3′, U6 forward, 5′-ATT GGA ACG ATA CAG AGA AGA TT-3′ and reverse, 5′-GGA ACG CTT CAC GAA TTT G-3′; ZEB1 forward 5′-CAG GCA GAT GAA GCA GGA TG-3′ and reverse 5′-CAG CAG TGT CTT GTT GTT GTA G-3′; β-actin forward 5′-GGC GGC ACC ACC ATG TAC CCT-3′ and reverse 5′-AGG GGC CGG ACT CGT CAT ACT-3′. The PCR conditions were: initial denaturation at 95°C for 3 min, followed by 40 cycles of 95°C for 15 s, 62°C for 30 s, and 72°C for 30 s.

Real-time PCR was performed using SYBR Green PCR Master Mix (Applied Biosystems) on an ABI 7300HT real-time PCR system (Applied Biosystems, Foster City, CA, USA). Standard curves were generated, and the relative amount of miR-150 or ZEB1 was normalized to the amount of U6 or β-actin, respectively. Relative quantification of target gene expression was evaluated using the 2^−△△CT^ method.

### Construction of expression vectors and cell transfection

The pcDNA_6.2-GW/EmGFP-miR plasmid vector (Invitrogen, Carlsbad, CA, USA) with a spectinomycin resistant gene was used to construct a miR-150 overexpressing plasmid. A 62 bp DNA fragment with mature miR-150 or a negative control (NC) mismatched sequence was chemically synthesized and added to this vector by Shanghai Shenggong Biotech (Shanghai, China).

EOC cells were harvested and plated onto 6-well plates with 70–80% confluence overnight before the transfection. The hsa-miR-150 vector and NC was transfected with Lipofectamin 2000 (Invitrogen, Carlsbad, CA, USA) at a concentration of 100 nM for 48 h at 37°C according to the manufacturer’s instruction.

### ZEB1 siRNA and cell transfection

ZEB1 siRNA (siRNA-ZEB1) and control siRNA (siRNA-NC) were purchased from Santa Cruz Biotechnology (Santa Cruz, CA, USA) and were transfected into EOC cells during the logarithmic growth phase using Lipofectamine 2000 liposome (Invitrogen Co., Carlsbad, CA, USA) at a concentration of 100 nM for 48 h at 37°C according to the manufacturer’s instruction. The expression level of ZEB1 was detected by Western blot to determine the interference effect for ZEB1.

### Western blot analysis

EOC cells were harvested 72 h after transient transfection and western blot analysis was performed to detect the expression levels of ZEB1 protein. Cells were lysed using RIPA buffer (50 mM Tris-HCl, pH 8.8, 150 mM NaCl, 1% NP-40, 1% sodium deoxycholate, 0.1% SDS). The proteins were resolved on an SDS denaturing polyacrylamide gel and then transferred onto a nitrocellulose membrane. Filters were blocked in PBS-Tween skim milk and probed with anti-ZEB1 antibody (dilution 1∶1000, Santa Cruz Biotechnology, Santa Cruz, USA) or probed with anti-β-actin antibody (Santa Cruz Biotechnology, Santa Cruz, USA). β-actin was used as an internal control for the normalization of candidate genes. The membranes were washed and incubated with horseradish peroxidase (HRP)-conjugated secondary antibodies. Protein expression was assessed by enhanced chemiluminescence and exposure to chemiluminescent film (Pierce Biotechnology).

### Luciferase reporter assay

The 3′-UTR of ZEB1 mRNA was cloned and inserted into the downstream of luciferse gene in pGL3/luciferase vector (Promega, Madison, WI, USA). The mutant 3′-UTR of ZEB1 mRNA was cloned using the wild type 3′-UTR as a template and inserted into pGL3/luciferase as described for the wild type 3′-UTR. EOC cells were cultivated in 24-well plates and were co-transfected with miR-150 mimics and pGL3-ZEB1-wt or pGL3-ZEB1-mut. At 48 h after transfection, EOC cells were harvested and luciferase activity was measured using the Dual-Luciferase Reporter Assay System (Promega, Madison, WI, USA) following the manufacturer’s instructions. The Renilla luciferase activities were used as an internal control. The experiments were performed independently in triplicate.

### In vitro cell proliferation assay

The in vitro cell proliferation of EOC cells transfected with hsa-miR-150 vector and NC vector was determined at 24, 48 and 72 h by using the CellTiter 96 AQueous One Solution Cell Proliferation Assay kit (Promega, Madison, WI) according to the manufacturer’s protocol. The absorbance at 490 nm was measured using a mQuant Universal Microplate Spectrophotometer (BioTek, Winooski, VT). Data are presented as the mean value for triplicate experiments.

### In vitro invasion assay

The invasion ability of EOC cells transfected with hsa-miR-150 vector and NC vector was tested in Matrigel coated cell culture chambers (8 µm pore size, Millipore, Billerica, MA, USA). EOC cells were transfected and cultured to confluence or near (>90%) confluence in 24-well dishes. Then, EOC cells were resuspended in 200 µl serum-free 1640 medium were placed into the upper chamber of the insert with Matrigel. Medium with 5% FBS was added into the lower chambers as a chemoattractant. After 24 h of incubation, cells remaining on the upper membrane were carefully removed. Cells that had invaded through the membrane were manually counted at 200× magnification from ten different fields of each filter. All experiments were done in triplicate.

### In vitro migration assay

The migration ability of EOC cells transfected with hsa-miR-150 vector and NC vector was tested in Corning transwell insert chambers. Briefly, 48 h after transfection, EOC cells were resuspended in 200 µl serum-free 1640 medium were placed into the upper chamber of the insert without Matrigel. Medium with 5% FBS was added into the lower chambers as a chemoattractant. After 24 h of incubation, cells remaining on the upper membrane were carefully removed. Cells that had migrated through the membrane were manually counted at 200× magnification from ten different fields of each filter. All experiments were done in triplicate.

### Statistical analysis

Data are expressed as mean ± S.E. Comparisons between groups were performed using the Kruskal- Wallis test for continuous variables and the χ^2^ test for categorical variables. The Kaplan-Meier method was used for survival analysis, and differences in survival were estimated using the log-rank test. A multivariate survival analysis was performed for all parameters that were significant in the univariate analyses using the Cox regression model. P<0.05 was considered significant.

## Results

### Downregulation of miR-150 in human EOC tissues

**Figure 1:**
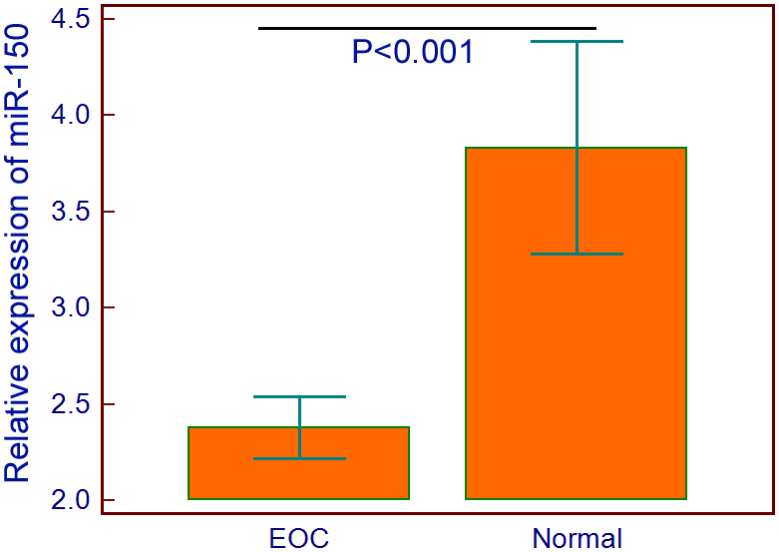
Expression levels of miR-150 in 100 EOC tissue specimens and 10 normal ovarian tissue specimens were detected by qRT-PCR and normalized to RNU6B. The results showed that the expression level of miR-150 in EOC tissues was significantly lower than that in normal ovarian tissues (EOC vs. Normal: 2.38±0.80 vs. 3.83±0.77, P<0.001).

Expression level of miR-150 in 100 EOC tissue specimens and 10 normal ovarian tissue specimens were detected by qRT-PCR and normalized to RNU6B. As a result, the expression level of miR-150 in EOC tissues was significantly lower than that in normal ovarian tissues (EOC vs. Normal: 2.38±0.80 vs. 3.83±0.77, P<0.001, Figure 1). The median value (2.36) of miR-150 expression in all EOC tissues detected by qRT-PCR was used as a cutoff point to classified 100 EOC patients into miR-150-low (n = 58, 58.00%) and miR-150-high (n = 42, 42.00%) expression groups.

### Downregulation of miR-150 associates with aggressive tumor progression of human EOC

Table 1 showed the associations between various clinicopathologic variables and miR-150 expression levels in tumor specimens from EOC patients. The EOC tissues with advanced clinical stage (III∼IV) more frequently showed low miR-150 expression than those with low clinical stage (I∼II, P = 0.005, Table 1). Additionally, miR-150 expression exhibited a trend that correlated with pathological grade (P = 0.02, Table 1). We found that miR-150 was aberrantly downregulated in tumor tissues with high pathological grade compared to those with low pathological grade. However, miR-150 expression was not associated with other clinicopathologic variables, including age, grade, histological type and residual tumor after surgery (all P>0.05).

### Downregulation of miR-150 associates with poor prognosis in patients with EOCs

**Figure 2:**
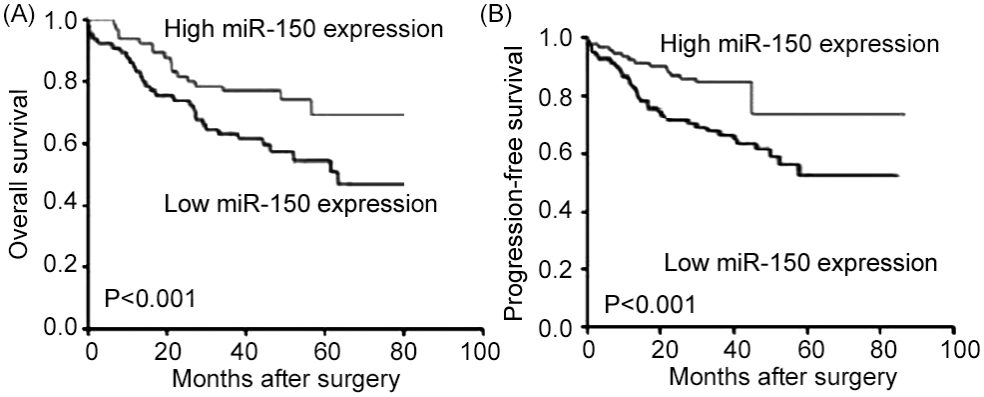
Kaplan–Meier overall (A) and progression-free (B) survival curves for epithelial ovarian cancer patients with high and low miR-150 expression. Epithelial ovarian cancer patients with low miR-150 expression had significantly shorter overall (P<0.001) and progression-free (P<0.001) survival than those with high miR-150 expression did.

OS and PFS curves were plotted according to the expression level of miR-150 in tumor specimens from EOC patients by the Kaplan-Meier method. As shown in Figure 2, EOC patients with low miR-150 expression had significantly shorter overall (P<0.001, Figure 2A) and progression-free (P<0.001, Figure 2B) survivals than those with high miR-150 expression did.

Univariate analysis with Cox proportional hazards model identified three prognostic factors: clinical stage, pathological grade and miR-150 expression. The other clinicopathological features, such as age, grade, histological type and residual tumor after surgery, were not statistically significant prognostic factors (Table 2). A multivariate analysis of the prognosis factors with a Cox proportional hazards model confirmed that clinical stage and miR-150 expression were two significant independent predictors of both OS and PFS in patients with EOC (Table 3).

**Table 2:** Univariate analysis: factors predicting overall and progression-free survival.

Characteristic	Groups	Overall survival		Progression-free survival	
		P value	Hazard Ratio (95% CI)	P value	Hazard Ratio (95% CI)
Age (years)	<56 vs. ≥56	NS	1.683 (0.721∼3.377)	NS	0.910 (0.639∼1.902)
Clinical stage	I∼II vs. III∼IV	<0.001	9.996 (1.928∼20.875)	0.006	6.396 (1.291∼13.763)
Pathological grade	1∼2 vs. 3	0.04	4.256 (1.032∼10.893)	NS	3.082 (1.006∼7.221)
Histological type	Serous vs. Non-serous	NS	6.826 (1.262∼13.812)	NS	4.206 (1.002∼8.816)
Residual tumor after surgery	<1 cm vs. ≥1 cm	NS	3.042 (1.001∼6.823)	NS	2.670 (0.823∼5.618)
miR-150 expression	Low vs. High	<0.001	10.369 (1.925∼21.962)	<0.001	9.128 (1.876∼19.598)

**Table 3:** Multivariate analysis: factors predicting overall and progression-free survival.

Characteristic	Overall survival		Progression-free survival	
	P value	Hazard Ratio (95% CI)	P value	Hazard Ratio (95% CI)
Clinical stage	0.01	5.668 (1.018–11.019)	0.03	4.972 (1.002∼9.682)
Pathological grade	NS	3.258 (0.822–6.692)	-	-
miR-150 expression	0.01	6.018 (1.036–12.238)	0.01	5.211 (1.022∼10.629)

### MiRNA-150 targets ZEB1 in EOC tissues

To determine how the downregulation in miR-150 expression might promote tumor progression, the candidate target genes of miR-150 were collected from miRTarBase (Release 4.5: Nov. 1, 2013; http://mirtarbase.mbc.nctu.edu.tw/), which has accumulated more than fifty thousand miRNA-target interactions (MTIs), which are collected by manually surveying pertinent literature after data mining of the text systematically to filter research articles related to functional studies of miRNAs [17], [18]. In the current study, we only selected the candidate target genes which were validated experimentally by reporter assay, western blot and quantitative real-time PCR. As a result, three genes, such as MYB, EGR2 and ZEB1, were selected candidate target genes of miR-150. In addition, RNAhybrid (Version 2.1, http://bibiserv.techfak.uni-bielefeld.de/rnahybrid/submission.html) was used to calculate the minimum free energy (MFE) of the duplex miRNA:mRNA [19]. The miRNA is hybridized to the target in an energetically optimal way. RNAhybrid was optimized to show the hybridization at the 3′UTR of the target genes. The duplex miRNA:mRNA with lower MFE is more stable than that with higher MFE. As a result, the MFE values of miR-150:MYB, miR-150:EGR2 and miR-150:ZEB1 were respectively –11.51 kcal/mol, –14.30 kcal/mol and –18.00 kcal/mol. The duplex miR-150:ZEB1 has the best MFE, therefore, we chose ZEB1 as a candidate target gene for miR-150 in the further validation experiments.

**Figure 3:**
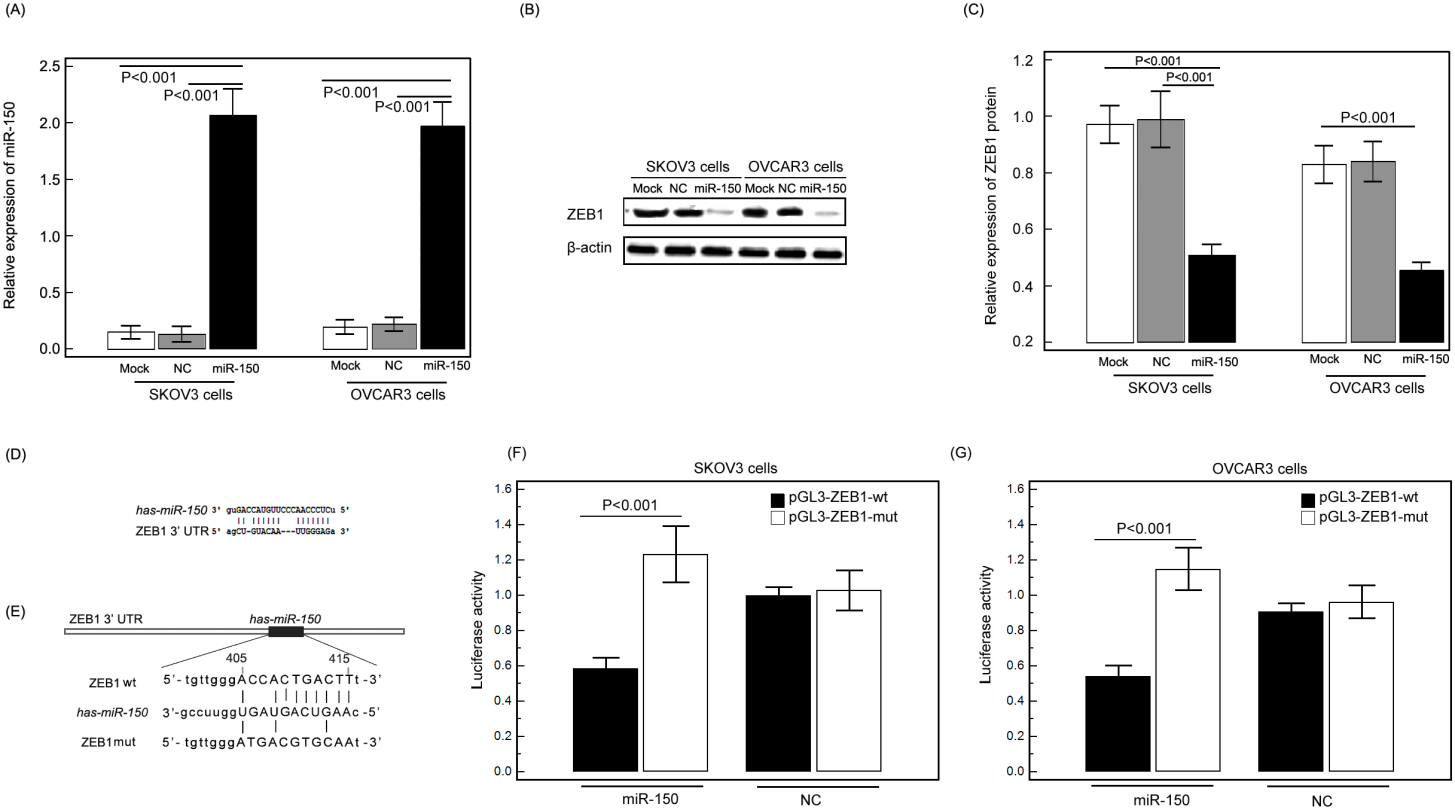
MicroRNA-150 (miR-150) targets Zinc Finger E-Box Binding Homeobox 1 (ZEB1) in epithelial ovarian cancer (EOC) cells. (A) Expression levels of miR-150 in SKOV3 and OVCAR3 cells by quantitative real-time PCR assay at 24 h post-transfection of miR-150 vector. (B∼C) The ZEB1 protein in SKOV3 and OVCAR3 cells by western blot at 24 h post-transfection of miR-150 vector. β-actin was used as an internal loading control. ‘NC’ refers to negative control vector. (D) miR-150 binding sites in the ZEB1 3′-UTR. (E) RNA sequence alignment showing the 3′-UTR of ZEB1 mRNA contains a complementary site for the seed region of miR-150. ZEB1 mut is a mutant with substitutions in the complementary region as a negative control. (F and G) Luciferase report assay was performed to confirm the miR-150 binding target. The luciferase activity was detected after co-transfection of pGL3-ZEB1-wt or pGL3-ZEB1-mut, miR-150 vector or negative control vector (NC) into SKOV3 and OVCAR3 cells.

In order to verify the candidate target of hsa-miR-150, we transfected EOC cells with hsa-miR-150 vector, negative vector (NC), and blank control culture medium (mock), respectively. At 24 h post-transfection, western blot analysis was performed and the results in Figure 3A∼B showed that the enforced expression of hsa-miR-150 led to a marked decrease in the expression levels of endogenous ZEB1 protein compared to EOC cells transfected with NC or mock (SKOV3 and OVCAR3 cell groups: both P<0.001). In addition, the luciferase reporter assay was performed by co-transfection of miR-150 and a luciferase reporter plasmid containing the 3′UTR of human ZEB1. According to miRTarBase (Release 4.5: Nov. 1, 2013; http://mirtarbase.mbc.nctu.edu.tw/), there are one validated binding site and two predicted binding sites for miR-150 in the ZEB1 3′UTR. In the current study, we only analyzed the validated binding site as shown in Figure 3D. To check whether a direct interaction is involved between miR-150 and its target oncogene ZEB1, we performed luciferase reporter assays (Figure 3E). We found that co-transfection of miR-150 along with the wild type 3′UTR of ZEB1 caused a significant decrease in luciferase activity compared to controls (Figure 3F and G).

### MiR-150 inhibits cell proliferation of EOC cells in vitro by targeting ZEB1

**Figure 4:**
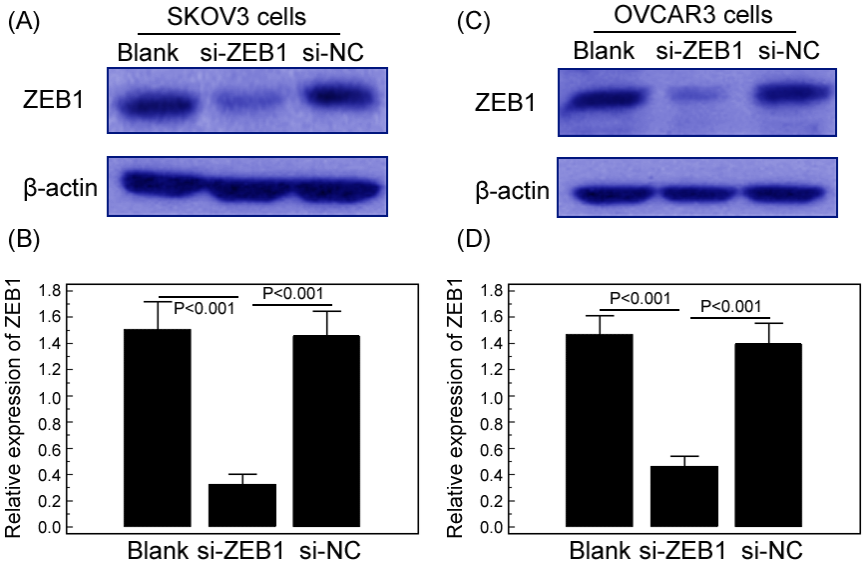
Down-regulation of Zinc Finger E-Box Binding Homeobox 1 (ZEB1) by siRNA interference. Western blot analysis was performed to detect the expression levels of ZEB1 protein in siRNA-ZEB1 transfected, siRNA-NC transfected and non-transfected (blank) SKOV3 (A and B) and OVCAR3 (C and D) cells. β-actin was used as an internal loading control.

**Figure 5:**
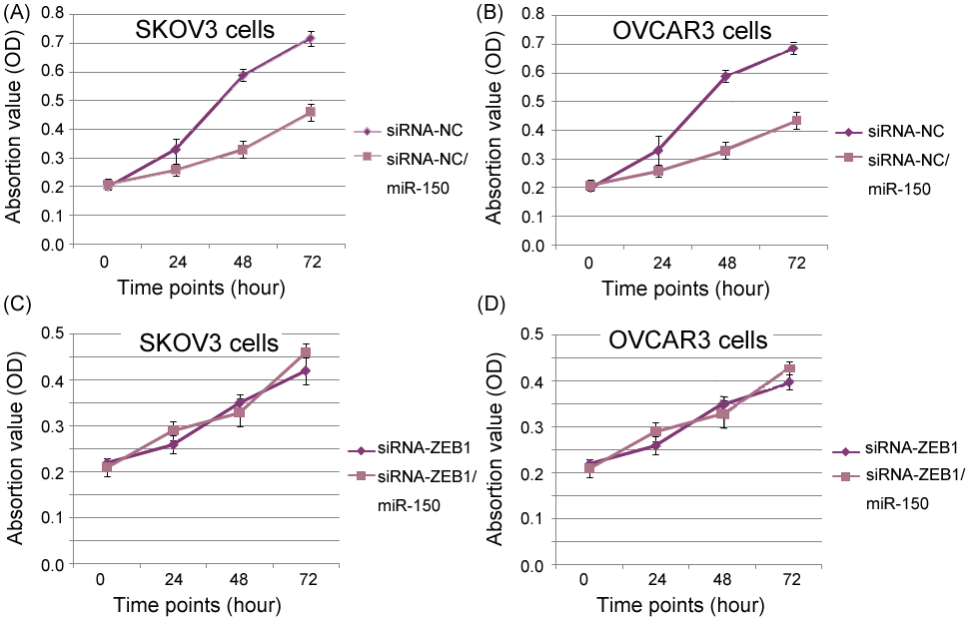
MicroRNA-150 (miR-150) inhibits cell proliferation of epithelial ovarian cancer (EOC) cells in vitro by targeting Zinc Finger E-Box Binding Homeobox 1 (ZEB1). (A and B) Growth curves of SKOV3 and OVCAR3 cells transfected with siRNA-NC, and SKOV3 and OVCAR3 cells transfected with siRNA-NC and miR-150 vector. (C and D) Growth curves of SKOV3 and OVCAR3 cells transfected with siRNA-ZEB1, and SKOV3 and OVCAR3 cells transfected with siRNA-ZEB1 and miR-150 vector.

To evaluate the effect of miR-150 on malignant phenotypes in EOC cells, we transfected siRNA-ZEB1 to specially suppress endogenous ZEB1 expression. As shown in Figure 4, the transfection of siRNA-ZEB1 could effectively inhibit the expression of ZEB1 protein in both SKOV3 and OVCAR3 cell lines (both P<0.001). In addition, we observed that the enforced expression of miR-150 significantly inhibited cell proliferation of SKOV3 and OVCAR3 cells, however, this alteration was reverted by the transfection of siRNA-ZEB1. As shown in Figure 5, the enforced expression of miR-150 significantly inhibited cell proliferation of SKOV3 and OVCAR3 cells transfected with siRNA-NC (both P = 0.006, Figure 5A and C) but failed to do so in SKOV3 and OVCAR3 cells transfected with siRNA-ZEB1 (both P>0.05, Figure 5B and D).

### MiR-150 inhibits EOC cells migration and invasion in vitro by targeting ZEB1

**Figure 6:**
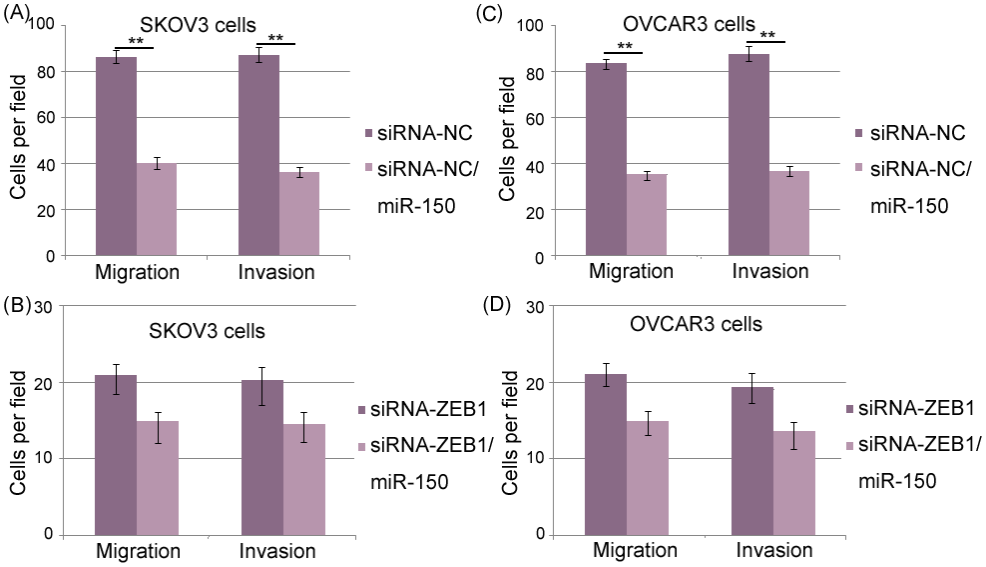
MicroRNA-150 (miR-150) inhibits epithelial ovarian cancer (EOC) cells migration and invasion in vitro by targeting Zinc Finger E-Box Binding Homeobox 1 (ZEB1). (A and C) Transwell migration assay and Matrigel invasion assay of EOC cells transfected with siRNA-NC, and EOC cells transfected with siRNA-NC and miR-150 vector. (B and D) Transwell migration assay and Matrigel invasion assay of EOC cells transfected with siRNA-ZEB1, and EOC cells transfected with siRNA-ZEB1 and miR-150 vector.

To further verify whether miR-150 inhibited the cell migration and invasion of EOC cell lines by targeting ZEB1, we also knocked down the expression of ZEB1 by the transfection of siRNA-ZEB1 in both SKOV3 and OVCAR3 cells. As shown in Figure 6, the enforced expression of miR-150 significantly inhibited the cell migration and invasion of SKOV3 and OVCAR3 cells transfected with siRNA-NC (both P = 0.01, Figure 6A and C) but failed to do so in SKOV3 and OVCAR3 cells transfected with siRNA-ZEB1 (both P>0.05, Figure 6B and D).

### Negative correlation between miR-150 and ZEB1 mRNA expression levels in human EOC tissues

**Figure 7:**
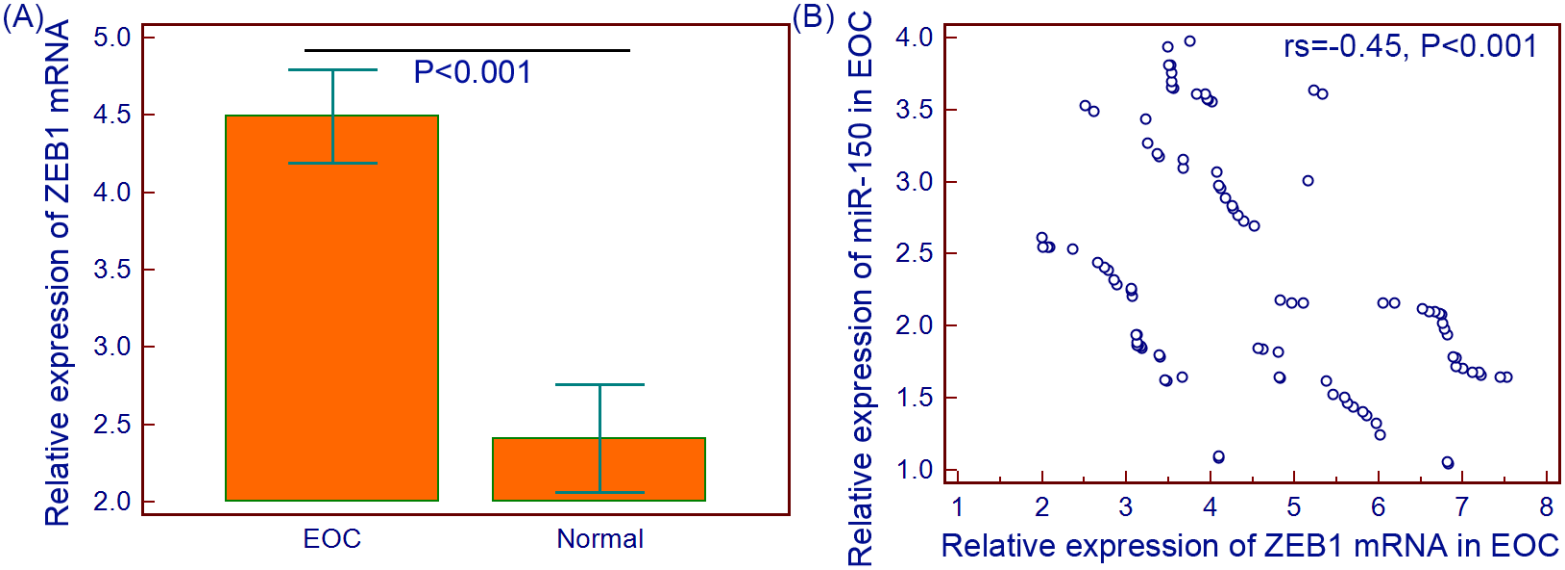
Expression levels of ZEB1 mRNA in 100 EOC tissue specimens and 10 normal ovarian tissue specimens were detected by qRT-PCR and normalized to β-actin. Expression level of ZEB1 mRNA in EOC tissues was significantly higher than that in normal ovarian tissues (EOC vs. Normal: 4.49±1.52 vs. 2.41±0.49, P<0.001, A). More interestingly, the Spearman Correlation analysis clearly showed negative correlation between miR-150 and ZEB1 mRNA expression in EOC tissues (rs = –0.45, P<0.001, B).

In order to evaluate the relationship between miR-150 and ZEB1 mRNA expression in EOC tissues, we further detected the expression levels of ZEB1 mRNA in 100 EOC tissue specimens and 10 normal ovarian tissue specimens by qRT-PCR and normalized to β-actin. As shown in Figure 7A, the expression level of ZEB1 mRNA in EOC tissues was significantly higher than that in normal ovarian tissues (EOC vs. Normal: 4.49±1.52 vs. 2.41±0.49, P<0.001, Figure 7A). More interestingly, the Spearman Correlation analysis clearly showed negative correlation between miR-150 and ZEB1 mRNA expression in EOC tissues (rs = –0.45, P<0.001, Figure 7B).

## Discussion

EOC is still a major gynecologic problem with low 5-year survival rate and seriously threatens human health due to distance metastases, despite routine surgery and chemotherapy. Growing evidence display the dysregulation of various miRNAs in EOC and imply their essential roles in tumorigenic processes, including cell proliferation, apoptosis and motility. Thus, revealing the molecular changes of miRNAs is crucial for overcoming this deadly disease. Previous studies have demonstrated that miR-150 is dramatically downregulated in human EOC tissues and patients’ serum compared to normal controls [14], [15]. However, the roles of miR-150 in initiation and progression of EOC and the downregulation of miR-150 expression in this cancer are still unclear. In the current study, we linked miR-150 to its target gene ZEB1, and demonstrated their involvements in regulating malignant phenotypes of ovarian cancer cells. We confirmed the key role of miR-150 as a tumor suppressor by directly and negatively targeting ZEB1 in EOC. The evidence for this comes from the following sources. First, we validated the downregulation of miR-150 in EOC tissues using a large cohort of EOC patients, and showed that low miR-150 expression level was much lower in highly aggressive EOC tissues. Second, the downregulation of miR-150 was identified as an independent prognostic marker for both overall and progression-free survivals of patients with EOC. Third, overexpression of miR-150 could dramatically inhibit the cell proliferation and motility of ovarian cancer cells in vitro and substantially suppress the protein expression of ZEB1. Forth, ZEB1 was identified as a direct target of miR-150, and knock-down of ZEB1 in ovarian cancer cells could mimic the inhibition of cell proliferation, migration and invasion by miR-150. Furthermore, we found a significantly negative correlation between miR-150 and ZEB1 mRNA expression in EOC tissues.

MiR-150 has proven to be an essential miRNA implicated in the development and the progression of various human malignancies. For instance, the decreased expression of miR-150 acts as an anti-apoptotic factor in human diffuse gastric cancer and adrenocorticotrophic hormone-secreting pituitary tumors [20]; Injection of miR-150-transduced mouse lymphoma cells into immunodeficient mice may produce fewer tumors than control cells [21]; Enforced expression of miR-150 may inhibit tumor cell growth in vitro and inhibit tumor growth in animal models through direct downregulation of DKC1 and AKT2, reduction of phosphorylated AKTser473/4 and an increase in tumor suppressors such as Bim and p53, leading to telomerase activation and immortalisation of cancer cells [22]; miR-150 expression is reduced in non-small cell lung carcinoma and was strongly associated with tumor stage, tumor size and patients’ survival as significantly low expression in advanced-stage, large-size and poor prognosis tumors was noted [13]. Decreased expression of miR-150 is also observed in esophageal squamous cell carcinoma and is indicated to be contributed to malignant potential, such as tumor depth, lymph node metastasis, lymphatic invasion, venous invasion, clinical staging, and poor prognosis [23]. In the current study, our data are consistent with these previous published studies that provide evidence for changes in miR-150 expression promoting tumor formation. We demonstrated that miR-150 was functionally involved in suppressing EOC cell growth, migration and invasion, which was supported by both cell culture studies and clinical data. In cell culture experiments, over-expression of miR-150 led to the decrease of EOC cell proliferation and cell motility. In clinical samples, miR-150 was dramatically down-regulated in EOC tissues with high clinical stage and high pathological grade compared with EOC tissues with low clinical stage and low pathological grade, and low miR-150 expression in tumors was associated with poor survival of patients with EOC.

More importantly, our identified miR-150 target was ZEB1, a member of the zinc finger family of proteins [24]–[26]. ZEB1 is one of the transcriptional inducer in the procedure of epithelial-mesenchymal transition (EMT) in cancer of epithelial origin, such as breast cancer, lung cancer, esophageal squamous cell carcinoma, gastric carcinoma, pancreatic cancer, cervical cancer, endometrial cancer and prostate cancer [23], [27]–[31]. Especially in EOC, Chen et al. [32] reported that downregulating ZEB1 expression with an expression vector-based small hairpin RNA (shRNA) targeting ZEB1 (shZEB1) in EOC SKOV3 cells could inhibit EMT of shZEB1-SKOV3 cells and block shZEB1-SKOV3 cell metastasis in vivo, suggesting its role in enhancing EMT in the EOC cells. They also observed that the shRNA-mediated down-regulation ZEB1 in SKOV3 cells could significantly decrease the tumor growth in the xenograft mice. Our data here showed the alteration of miR-150 in ovarian cancer cells led to opposite change of ZEB1, highlighting their negatively regulation. Meanwhile, ZEB1 mRNA 3′UTR bears a binding site of miR-150. We also confirmed that miR-150 could block migration and invasion of ovarian cancer cells by targeting ZEB1, which would constitute a promising target for rational cancer therapy.

Taken together, these findings offer the convincing evidence that aberrant expression of miR-150 may play a role in tumor progression and prognosis in patients with EOC. Moreover, our data reveal that miR-150 may function as a tumor suppressor and modulate EOC cell proliferation, and invasion by directly and negatively regulating ZEB1, implying the re-expression of miR-150 might be a potential therapeutic strategy for EOC.
